# The relevance of transdiagnostic shared networks to the severity of symptoms and cognitive deficits in schizophrenia: a multimodal brain imaging fusion study

**DOI:** 10.1038/s41398-020-0834-6

**Published:** 2020-05-18

**Authors:** Shile Qi, Juan Bustillo, Jessica A. Turner, Rongtao Jiang, Dongmei Zhi, Zening Fu, Thomas P. Deramus, Victor Vergara, Xiaohong Ma, Xiao Yang, Mike Stevens, Chuanjun Zhuo, Yong Xu, Vince D. Calhoun, Jing Sui

**Affiliations:** 1 Tri-institutional Center for Translational Research in Neuroimaging and Data Science (TReNDS), Georgia State University, Georgia Institute of Technology, Emory University, Atlanta, GA 30303 USA; 2 Department of Psychiatry, University of New Mexico, Albuquerque, NM 87131 USA; 3 Department of Psychology, Georgia State University, Atlanta, GA 30302 USA; 4 Brainnetome Center and National Laboratory of Pattern Recognition, Institute of Automation, Chinese Academy of Sciences, 100190 Beijing, China; 5 University of Chinese Academy of Sciences, 100190 Beijing, China; 6 Psychiatric Laboratory and Mental Health Center, The State Key Laboratory of Biotherapy, West China Hospital of Sichuan University, 610041 Chengdu, China; 7 Huaxi Brain Research Center, West China Hospital of Sichuan University, 610041 Chengdu, China; 8 Olin Neuropsychiatry Research Center, Hartford, CT 06106 USA; 9 Department of Psychiatry, Nankai University Affiliated Anding Hospital, 300222 Tianjin, China; 10 Department of Humanities and Social Science, Shanxi Medical University, 030001 Taiyuan, China; 11 Chinese Academy of Sciences Center for Excellence in Brain Science, Institute of Automation, 100190 Beijing, China

**Keywords:** Autism spectrum disorders, Schizophrenia, Addiction, Depression, ADHD

## Abstract

Schizophrenia (SZ) is frequently concurrent with substance use, depressive symptoms, social communication and attention deficits. However, the relationship between common brain networks (e.g., SZ vs. substance use, SZ vs. depression, SZ vs. developmental disorders) with SZ on specific symptoms and cognition is unclear. Symptom scores were used as a reference to guide fMRI-sMRI fusion for SZ (*n* = 94), substance use with drinking (*n* = 313), smoking (*n* = 104), major depressive disorder (MDD, *n* = 260), developmental disorders with autism spectrum disorder (ASD, *n* = 421) and attention-deficit/hyperactivity disorder (ADHD, *n* = 244) respectively. Common brain regions were determined by overlapping the symptom-related components between SZ and these other groups. Correlation between the identified common brain regions and cognition/symptoms in an independent SZ dataset (*n* = 144) was also performed. Results show that (1): substance use was related with cognitive deficits in schizophrenia through gray matter volume (GMV) in anterior cingulate cortex and thalamus; (2) depression was linked to PANSS negative dimensions and reasoning in SZ through a network involving caudate-thalamus-middle/inferior temporal gyrus in GMV; (3) developmental disorders pattern was correlated with poor attention, speed of processing and reasoning in SZ through inferior temporal gyrus in GMV. This study reveals symptom driven transdiagnostic shared networks between SZ and other mental disorders via multi-group data mining, indicating that some potential common underlying brain networks associated with schizophrenia differently with respect to symptoms and cognition. These results have heuristic value and advocate specific approaches to refine available treatment strategies for comorbid conditions in schizophrenia.

## Introduction

Schizophrenia (SZ) is a chronic, severe mental disorder affecting up to 1% of the world’s population and substantially contributing to the global burden of mental disorders^1^. In categorical diagnostic systems, such as the ICD/DSM^2^, symptomological overlap across diagnostic categories is highly prevalent, as is frequently seen across SZ, bipolar disorder^3^, depression, and substance use disorders^4^. Evidence of such transdiagnostic overlap has also been reported in brain structural^5,6^ and genetic^7–9^ studies. Specifically, SZ diagnosis is frequently concurrent with substance use, such as smoking and drinking^10,11^, showing a prevalence of 50%, for example. Neuroimaging findings comparing substance use disorders and SZ show that they both involve reward network dysfunction related with impulsive control^10^.

Depressive symptoms are also very common throughout the course of SZ, with 25% meeting the diagnostic criteria for major depressive disorder (MDD) at some time in their lives, and the prevalence of any type of depressive disorder has been reported to be around 40%^10^. Mood symptoms in depression have shown specific overlap with negative symptoms in SZ^12^. SZ and autism spectrum disorder (ASD) also share behavioral symptoms such as social withdrawal and communication impairment (Ford et al.^13^). Interestingly, childhood onset schizophrenia and autism were historically once considered to be the same disorder expressed at different developmental periods, with autism manifesting as an earlier phase of schizophrenia. Attentional and related cognitive deficits are prevalent in both SZ and other developmental disorders such as attention-deficit/hyperactivity disorder (ADHD)^14^.

Hence, at a phenomenological level, because schizophrenia shares similar behavioral and symptom characteristics with substance use, depression, and neurodevelopmental disorders, some neuroimaging studies have taken a variety of approaches to identify potential similarities in their shared brain networks between SZ and substance use^15,16^, SZ and ASD^17^, SZ and depression^12^ separately. To date, there are few studies that have evaluated the common neuroimaging findings between SZ and other mental disorders using the same methodological and analytic approach in one study. Most existing neuroimaging studies have compared brain structure or function in patients with a single, specific diagnosis with relevant control participants, or were restricted to single imaging modality, i.e., GM^15^, population based demographic reviews^18^, or via meta-analysis^5^. Although these studies provide useful hints regarding the shared structures or functions underlying such shared symptoms or behaviors, it is still unclear how these shared brain networks may relate with SZ on specific symptoms and cognition differently. There is increasing imaging and genetic evidence showing that there are common brain regions (anterior cingulate cortex and insular in gray matter)^5^, common neurocognitive networks^19^, and common genetic underpinnings^20^ shared among different psychiatric disorders, conceptually due to the disorder’s shared symptoms and behavioral deficits. Here, we hypothesis that the common brain structures and functions among smoking, drinking, MDD, ASD, ADHD and SZ may have certain varying relationships with symptoms and cognition in SZ, and we examine this directly in a multi-study aggregation of functional-structural imaging and clinical measures.

Multimodal brain imaging^21,22^ data of SZ (*n* = 238), drinkers (*n* = 313), smokers (*n* = 104), MDD (*n* = 260), ASD (*n* = 421), and ADHD (*n* = 244) were collected across multiple studies^23–26^. We used specific symptom scores for each disorder as a reference to guide resting-state functional MRI (fractional amplitude of low frequency fluctuations, fALFF) and structural MRI (gray matter volume, GMV) fusion analysis to identify the multimodal brain networks that were associated with symptom scores in Center for Biomedical Research Excellence (COBRE) SZ, drinkers, smokers, MDD, ASD, and ADHD separately. FALFF can directly provide information of the amplitude of brain activity of each brain region within a network, i.e., reveals the BOLD signals change of the regional spontaneous activity. Significantly decreased fALFF throughout the brain in the cortical edge, and significantly increased ALFF in subcortical regions were observed in SZ^27^. Then the common brain regions were determined by overlapping the identified symptom-related components across the different diagnostic groups. Finally, correlation analysis between the identified common brain regions and the cognition/symptom was performed to evaluate the relationship of these brain regions with cognition or symptoms specifically in Function Biomedical Informatics Research Network (FBIRN) SZ. In general, we identify shared brain abnormalities in a subgroup of schizophrenia patients (COBRE, *n* = 94), and calculate the association between shared brain abnormalities in relation to cognitive functions and symptoms in another subgroup of schizophrenia patients (FBIRN, *n* = 144). Our overall hypothesis was that some of the shared networks between schizophrenia and several co-morbid disorders, would be particularly relevant to the severity of symptoms and cognitive deficits in schizophrenia. For this investigation, we focused on identifying 1) the common brain networks between SZ and substance use (drinking and smoking), SZ and depression (MDD), and SZ and developmental disorder (ASD and ADHD); 2) the relationship between the above identified common brain networks with cognition and symptoms in SZ.

## Methods and materials

### Participants

The data from subjects with SZ (*n* = 238) were aggregated from FBIRN and COBRE, studies as described in^23^. The SZ subjects had no current or past history of other psychiatric or neurological illness. MDD (*n* = 260) were recruited from the West China Hospital of Sichuan, Henan Mental Hospital of Xinxiang, Beijing Anding Hospital and First Affiliated Hospital of Zhejiang^25,28^. ADHD (*n* = 244) data was obtained from the ADHD-200 project (http://fcon_1000.projects.nitrc.org/indi/adhd200/index.html). ASD (*n* = 421) were obtained from the Autism Brain Imaging Data Exchange (ABIDE II)^29,30^. Diagnosis of SZ, MDD, ADHD and ASD were based on Structured Clinical Interview for DSM-IV in all of these samples. Symptom scores from the Positive and Negative Syndrome Scale (PANSS), Hamilton Depression Rating Scale (HAMD), Autism Diagnostic Interview-Revised (ADIR) and ADHD Rating Scale IV (ADHD-RS) (measures of inattentive/impulsive behaviors) were available for the SZ, MDD, ASD and ADHD samples, respectively. The severity of alcohol use of drinkers (*n* = 313) in the substance abuse sample was assessed with the Alcohol Use Disorder Identification Test (AUDIT). Nicotine dependence levels of smokers (*n* = 104) were assessed using the Fagerstrom Tolerance Questionnaire (FTQ). These substance use subjects, MDD, ASD, and ADHD had no current or past history of SZ. Detailed demographic information is provided in Table 1. The PANSS total, AUDIT, FTD, HAMD, ADIR and inattentive/impulsive were used as the reference for SZ, drinking, smoking, MDD, ASD, and ADHD group fusion, respectively (one measure per group).

**Table 1:** Demographic information.

Type	Number	Age	Gender	Symptom	R1	R2	*P* value
SZ	*n* = 238	37.4 ± 12.1	183 M	57.4 ± 14.8	0.9	0.05	0.04
Drinkers	*n* = 313	32.0 ± 9.8	219 M	19.0 ± 7.7	8.2e−16	0.9	0.9
Smokers	*n* = 104	26.4 ± 4.6	79 M	6.1 ± 3.4	0.01	0.60	0.6
MDD	*n* = 260	32.8 ± 11.0	99 M	19.3 ± 7.3	0.63	0.73	0.8
ASD	*n* = 421	13.5 ± 5.6	421 M	3.0 ± 1.4	0.003	NA	NA
ADHD	*n* = 244	11.3 ± 3.2	180 M	141.1 ± 18.0	0.043	0.051	0.06

R1 column means correlation between age and specific symptom scores (PANSS total, AUDIT, FTD, HAMD, ADIR, and inattentive/impulsive for SZ, drinking, smoking, MDD, ASD, and ADHD group, respectively), *p* values were listed.

R2 column means correlation between gender and specific symptom scores (PANSS total, AUDIT, FTD, HAMD, ADIR, and inattentive/impulsive for SZ, drinking, smoking, MDD, ASD, and ADHD group, respectively), *p* values were listed.

*P* value column means gender difference of symptom scores.

Cognitive measures for the FBIRN SZ sample were obtained from testing with the Computerized Multiphasic Interactive Neuro-cognitive System (CMINDS)^31^. The CMINDS includes computerized neuropsychological tasks that are structurally and functionally similar to standard paper-and-pencil neuropsychological tasks and allows for immediate electronic raw data capture and automated scoring of test results. The CMINDS-based cognitive domains include: Speed of Processing, Attention/Vigilance, Working Memory, Verbal Learning, Visual Learning, Reasoning/Problem Solving. A CMINDS composite score was defined as the mean of all six normalized domain scores.

### Multimodal imaging preprocessing

Multimodal brain imaging of resting-state fMRI and sMRI were available from each participant who met inclusion criteria. The fMRI data was preprocessed using statistical parametric mapping (SPM12, http://www.fil.ion.ucl.ac.uk/spm/) in the MATLAB 2018 environment. We performed rigid body motion correction using SPM to correct subject head motion, followed by the slice-timing correction to account for timing difference in slice acquisition. The fMRI data were subsequently warped into the standard Montreal Neurological Institute (MNI) space using an echo planar imaging (EPI) template and were resampled to 3 × 3 × 3 mm^3^ isotropic voxels. The resampled fMRI images were further smoothed using a Gaussian kernel with a full width at half maximum (FWHM) = 6 mm. Then for each voxel, six rigid body head motion parameters, white matter (WM) signals, and cerebrospinal fluid (CSF) signals were regressed out using linear regression. Finally, to calculate fALFF^32^, the sum of the spectral amplitude values in the 0.01 to 0.08 Hz low-frequency power range was divided by the sum of the amplitudes over the entire detectable power spectrum (range: 0–0.25 Hz).

The structural data T1 images were preprocessed through an automated SPM12 pipeline. Tissue classification, bias correction, image registration, and spatial normalization were automatically performed using voxel based morphometry (VBM) in SPM12, wherein the above steps are integrated into a unified model^33^. Modulated GM segmentations, which produces an estimation of GMV, are then smoothed using a Gaussian kernel with a full width at half maximum of 6 mm.

Next, each modality was reshaped into a feature matrix with columns representing voxels and rows representing subjects. Age and data acquisition sites were regressed out for ASD, drinkers and smokers; age, gender and sites were regressed out for ADHD; and age, gender and sites were regressed out for SZ, prior to fusion analysis, according to Table 1. Finally, the obtained feature matrices were normalized to have the same average sum of squares (computed across all subjects and all voxels for each modality) to ensure all modalities had the same range of values in each group.

### Study design

**Fig. 1:**
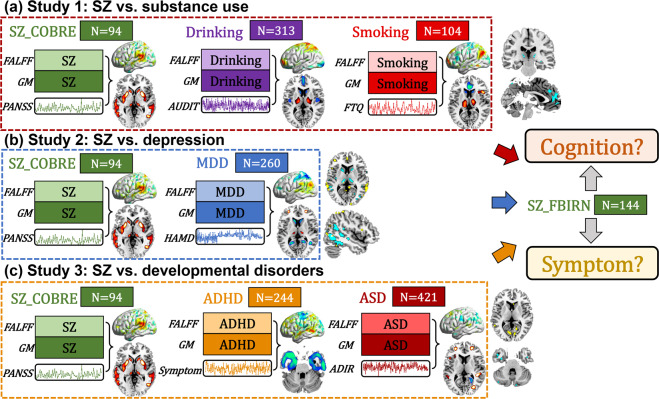
Flowchart of the study design. The study design includes three sections: (**a**) Study 1: SZ_COBRE vs. substance use, (**b**) Study 2: SZ_COBRE vs. depression, (**c**) Study 3: SZ_COBRE vs. developmental disorders. First, we identify specific symptom-associated multimodal components for COBRE SZ, drinking, smoking, MDD, ADHD and ASD groups separately. Then the common brain regions were determined by overlapping the derived symptom-related components of different diagnostic groups versus COBRE SZ (Study 1–3). Finally, correlation analysis was performed to evaluate how these identified brain regions associate with particular cognitive or symptomatic measures in an independent SZ dataset (FBIRN), which also test the replicability of our findings.

According to the two goals stated in the introduction, we performed a systematic, data-driven analysis as described in Fig. 1. Symptom scores were used as a reference to guide a group specific two-way MRI (fALFF+GM) fusion analyses for COBRE SZ (PANSS total), drinking (AUDIT), smoking (FTQ), MDD (HAMD), ADHD (total score of inattentive and impulsivity) and ASD (ADIR), respectively. Then the common regions were determined by overlapping the resulting components between COBRE SZ and different diagnostic groups (SZ_COBRE vs. substance use, SZ_COBRE vs. depression and SZ_COBRE vs. developmental disorders). Finally, a correlation analysis between the identified common brain regions and cognition (including Speed of Processing, Attention/Vigilance, Working Memory, Verbal Learning, Visual Learning, Reasoning/Problem Solving and composite) or symptom (7 negative scale, 7 positive scale and 14 general psychological scale, https://en.wikipedia.org/wiki/Positive_and_Negative_Syndrome_Scale) was performed to evaluate the relationship of these brain regions with cognition or symptoms particularly for FBIRN SZ. Note that the SZ data were separated into two subgroups: COBRE is used for the discovery cohort and FBIRN for validation in order to avoid the circular analysis.

Specifically, the preprocessed multimodal MRI features were jointly analyzed by a fusion-with-reference model called “MCCAR + jICA” (http://trendscenter.org/software/fit/, multi-site canonical correlation analysis with reference + joint independent component analysis)^34^, a data-driven analysis for identifying targeted brain regions associated with symptom scores^22^. Subject-wise symptom scores were used as a reference to jointly decompose fALFF and GM volume to investigate symptom-associated fALFF-GM covarying multimodal patterns in COBRE SZ, drinking, smoking, MDD, ADHD, and ASD groups individually. Note that “MCCAR + jICA” can simultaneously maximize the inter-modality covariation and correlations of neuroimaging components with symptoms. As a result, a joint multimodal component(s) which is correlated with symptoms are identified. To generate comparable components among different psychiatric groups, we used the same component number (IC = 50) for each group’s fusion analysis. The common brain regions across the different groups were identified by overlapping the thresholded symptom-related component among (1) SZ_COBRE vs. drinking vs. smoking, (2) SZ_COBRE vs. MDD, (3) SZ_COBRE vs. ASD vs. ADHD for fALFF and GM, respectively. The correlations were conducted between the mean fALFF or mean GM (the common brain regions were used as ROIs) and cognition or symptom scores in FBIRN SZ group only.

### Ethics statement

The studies involved in this analysis were approved by the local ethics committees and adhered to the Declaration of Helsinki, and the written informed consent was obtained from all subjects.

## Results

### Components related with PANSS in SZ

SZ related joint components were identified, which correlate with total PANSS scores (fALFF: *r* = −0.59, *p* = 4.6e−10*; GM: *r* = −0.61, *p* = 7.6e−11*), as shown in Supplementary Fig. 1. Asterisk (*) signifies false discovery rate correction (FDR) for multiple comparisons. Spatial maps were transformed into *Z* scores and are visualized at |Z| > 2 in Supplementary Fig. 1a. The positive/negative brain regions (red/blue) indicate positive/negative correlation with PANSS in fALFF or GMV, i.e., red GM volumes in the identified brain areas correlate with more severe PANSS total. SZ related multimodal patterns include positive brain fALFF in middle temporal gyrus (MTG) and negative fALFF in thalamus and lingual gyrus, accompanied with positive GM volume in striatum, thalamus, MTG, inferior temporal gyrus (ITG), anterior cingulate cortex (ACC), and hippocampus.

### Substance use related with cognitive deficits in SZ

**Fig. 2:**
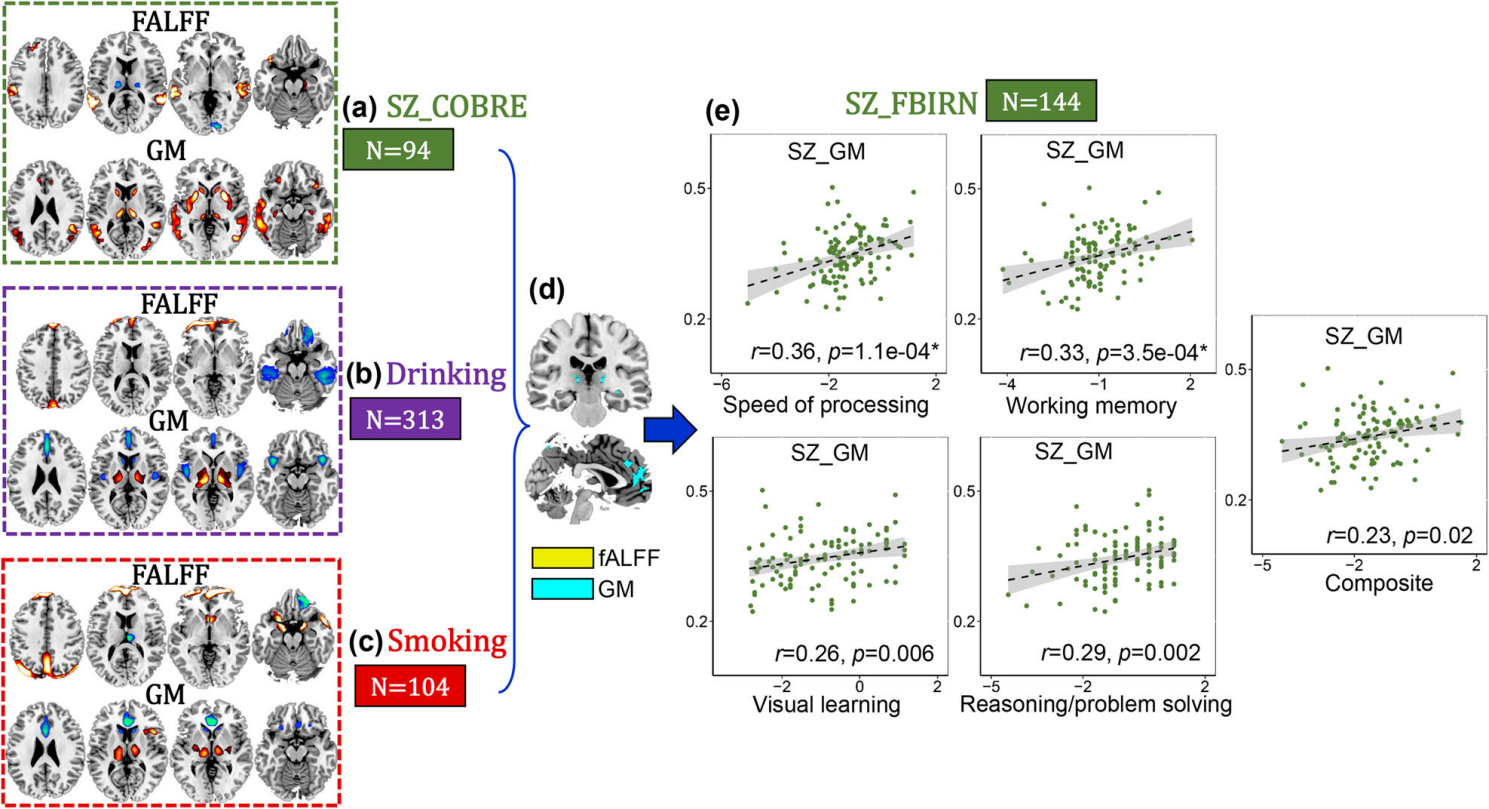
SZ vs. substance use. ACC-thalamus in GMV (**d**) are the common brain regions among COBRE SZ (**a**), drinking (**b**) and smoking (**c**), and are correlated with only cognitive deficits in FBIRN SZ (**e**).

Drinking (Fig. 2b) and smoking (Fig. 2c) related joint components were also identified, which correlate with AUDIT (fALFF: *r* = 0.46, *p* = 5.9e−18*, GM: *r* = 0.58, *p* = 2.8e−29* for drinking group and FTQ scores (fALFF: *r* = 0.50, *p* = 5.8e-08*, GM: *r* = 0.40, *p* = 2.9e−05*) for smoking group, as displayed in Supplementary Figs. 2, 3. After overlapping the drinking and smoking component patterns, ACC and thalamus in GMV were identified as common brain areas between substance use and SZ (Fig. 2d). The threshold used for identifying regions correlated with symptoms in different diagnostic groups are the same, i.e., |Z| > 2. The regions positively and negatively correlated with symptoms were treated equally in defining the transdiagnostic affected brain regions. Correlation analysis show that substance use related reward ACC-thalamus patterns are correlated with cognitive deficits (including speed of processing *r* = 0.36, *p* = 1.1e−04*, working memory *r* = 0.33, *p* = 3.5e−04*, visual learning *r* = 0.26, *p* = 0.006, reasoning *r* = 0.29, *p* = 0.002 and composite *r* = 0.23, *p* = 0.02) in FBIRN SZ but not PANSS symptoms (*p* > 0.05).

### Depression related with both cognition and negative PANSS in SZ

**Fig. 3:**
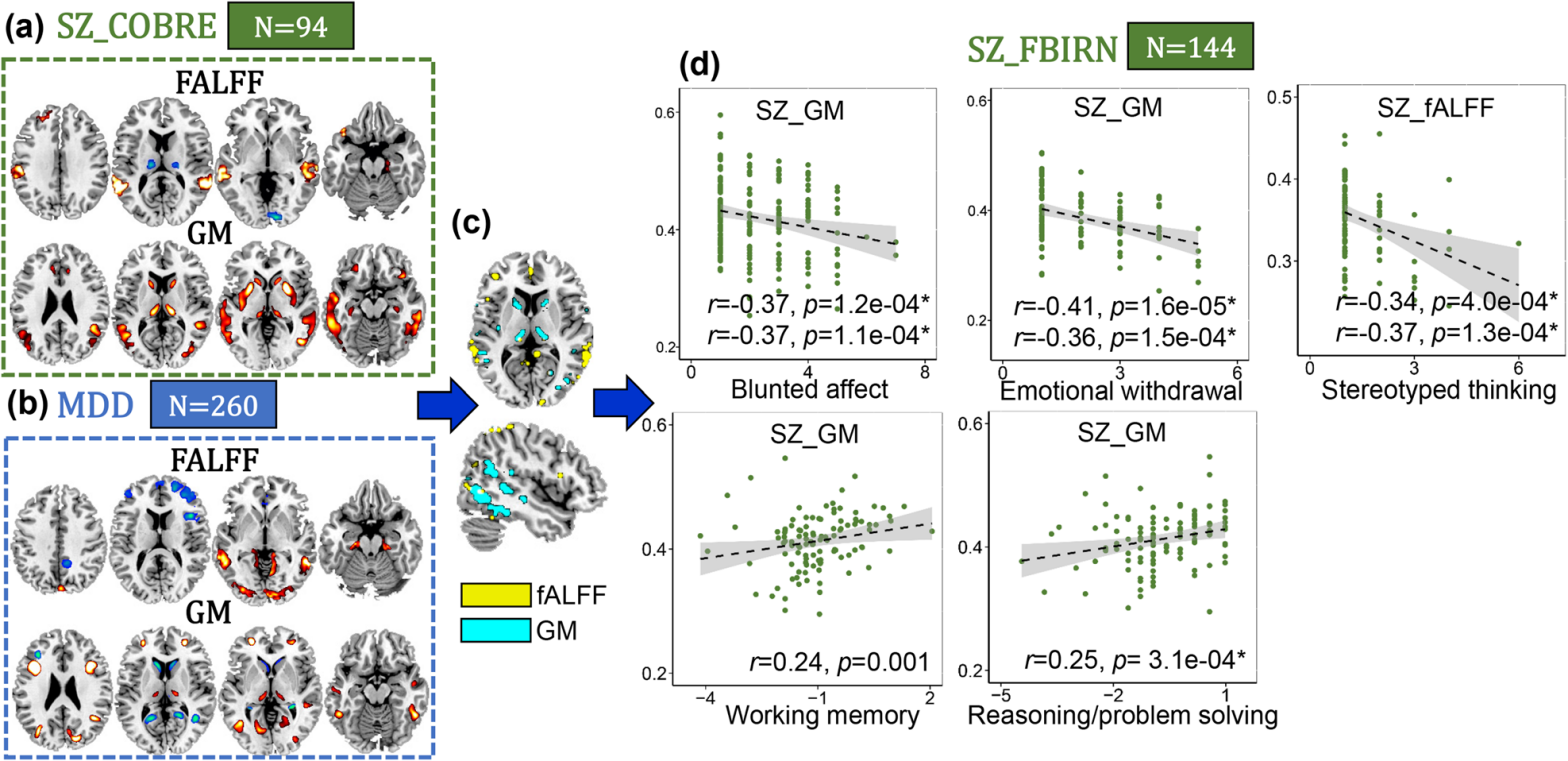
SZ vs. MDD. Caudate-thalamus-middle/inferior temporal gyrus (**c**) are the common brain regions between COBRE SZ (**a**), and MDD (**b**), and are correlated with both PANSS negative domains (blunted affect, emotional withdrawal and stereotyped thinking) and cognitive deficits (working memory and reasoning) in FBIRN SZ (**d**). For the discrete values in (**d**), Spearman correlation was also calculated (the second correlation value in each subfigure **d**).

MDD (Fig. 3b) related joint components correlate with HAMD scores (fALFF: *r* = 0.42, *p* = 4.3e−12*, GM: *r* = 0.41, *p* = 1.6e−11*) are shown in Supplementary Fig. 4. After overlapping with COBRE SZ pattern, middle and inferior temporal gyrus (MI_TG) and caudate-thalamus are the common GMV brain areas between MDD and SZ (Fig. 3c). Correlation analysis show that depression related patterns are correlated with both cognitive deficits (working memory *r* = 0.24, *p* = 0.001 and reasoning *r* = 0.25, *p* = 3.1e−04*) and PANSS negative dimensions (blunted affect *r* = −0.37, *p* = 1.2e−04*, emotional withdrawal *r* = −0.41, *p* = 1.6e−05* and stereotyped thinking *r* = −0.34, *p* = 4.4e−04*) in FBIRN SZ.

### Developmental disorders related with PANSS general and cognition in SZ

**Fig. 4:**
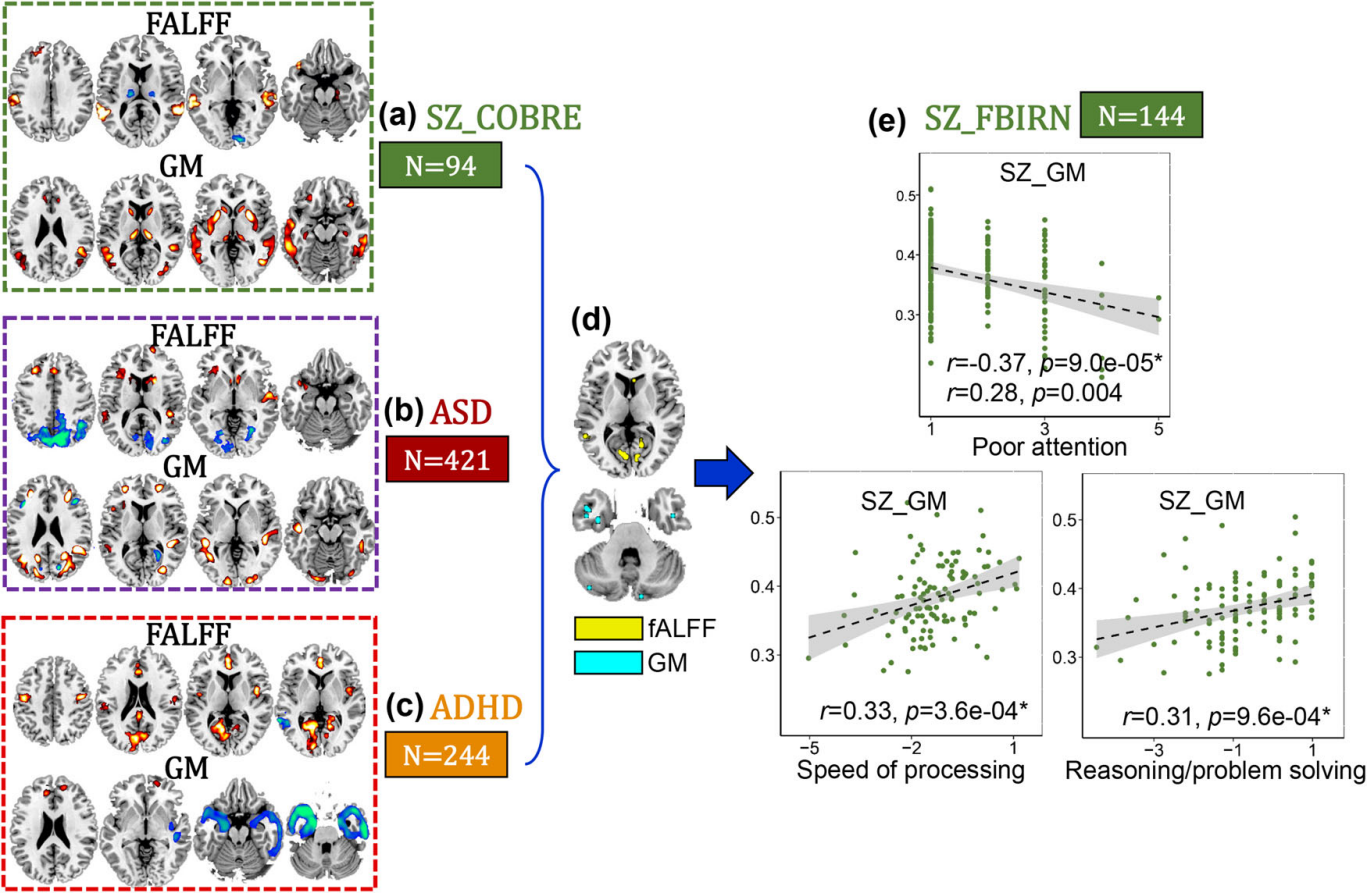
SZ vs. ASD and ADHD. Inferior temporal gyrus and lingual gyrus (**e**) are the common brain region among COBRE SZ (**a**), ASD (**b**), and ADHD (**c**), and are correlated with PANSS general (poor attention) and cognitive (speed of processing and reasoning) dimensions in FBIRN SZ (**e**). For the discrete values in **e**, Spearman correlation was also calculated (the second correlation value in each subfigure **e**).

ASD (Fig. 4c) and ADHD (Fig. 4d) related joint components correlated with symptom scores (fALFF: *r* = 0.37, *p* = 1.5e−09*, GM: *r* = 0.46, *p* = 3.2e−14* for ASD group and fALFF: *r* = 0.42, *p* = 5.4e−10*, GM: *r* = 0.29, *p* = 2.6e−05* for ADHD group) are shown in Supplementary Fig. 5, 6. After overlapping with COBRE SZ pattern, ITG in GMV and lingual gyrus in fALFF are the common brain areas among ASD, ADHD and SZ (Fig. 4d). Correlation analysis show that developmental disorder related patterns are associated with PANSS general (poor attention *r* = −0.37, *p* = 9.0e−05*) and cognitive dimensions (speed of processing *r* = 0.33, *p* = 3.6e−04* and reasoning *r* = 0.31, *p* = 9.6e−04*) dimensions in FBIRN SZ.

## Discussion

**Fig. 5:**
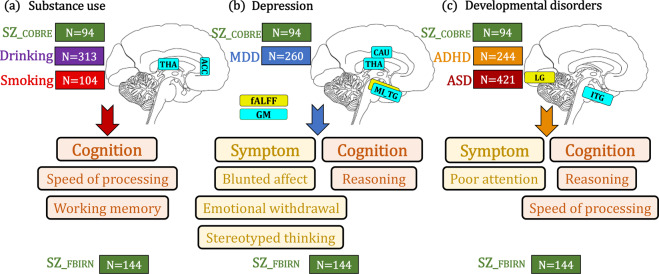
Summary on the relationships between schizophrenia and other mental disorders. **a** ACC-thalamus reward pattern in GMV are common between COBRE SZ and substance use, which correlate with cognitive deficits in FBIRN SZ especially with speed of processing and working memory domains. **b** Caudate-thalamus-MI_TG in GMV are common between COBRE SZ and depression, which correlate with both PANSS negative dimensions (including blunted affect, emotional withdrawal and stereotyped thinking) and cognition (reasoning) in FBIRN SZ. **c** ITG-lingual gyrus pattern in GMV and fALFF are common between SZ and DD, which correlate with PANSS general (poor attention) and cognition (speed of processing and reasoning). THA is thalamus; CAU is caudate; ACC is anterior cingulate cortex; MI_TG is middle and inferior temporal gyrus; LG is lingual gyrus; ITG is inferior temporal gyrus.

This study attempts to contribute to the understanding of the relationship between schizophrenia and drinking, smoking, depression, ASD and ADHD. Three key points are noteworthy (Fig. 5). First, substance use was related with cognitive deficits (speed of processing and working memory) in schizophrenia through GMV in ACC-thalamus reward circuit. Second, depression was linked to PANSS negative dimensions (blunted affect, emotional withdrawal and stereotyped thinking) and cognition (reasoning) in SZ through a network involving caudate-thalamus-middle/inferior temporal gyrus in GMV and fALFF. Third, developmental disorders correlate with PANSS general (poor attention) and cognitive dimensions (speed of processing and reasoning) in SZ through GMV in ITG.

The use of alcohol and/or illicit drugs in patients with schizophrenia is a remarkably common phenomenon. Our study found that ACC-thalamus circuit is common between SZ and substance use which associate with a core cognitive deficit in schizophrenia, especially in speed of processing and working memory. Researchers have found the ACC dysfunction among people with schizophrenia regardless of whether they smoked or not, as well as among the close relatives of people with schizophrenia^5^. Several ACC related neural circuits were also less active among individuals with severe nicotine use disorder, suggesting that this brain area is impaired in both schizophrenia and nicotine dependence. High-order cognitive domains, such as working memory in SZ has been associated with abnormal thalamic activation^35^. Our results suggest that ACC-thalamus dysfunction in GMV as detected among drinking, smoking and SZ may represent the common neural foundation in SZ and substance use, which is related with cognitive impairment in SZ, especially the speed of processing and working memory. Thus, we hypothesize that substance use could contribute to working memory and speed of processing deficits in SZ through affecting the ACC-thalamus gray matter structure. This hypothesis should be examined in a longitudinal MRI study of substance, using SZ patients before and after smoking or drinking cessation therapy. We would expect that SZ patients who successfully quit such addiction would show normalization in ACC-thalamus gray matter, as well as improvement in working memory and processing speed. Hence, our findings have heuristic value to guide the refinement of available treatments.

The thalamus is involved in the bidirectional flow of neuronal signals between cortical and subcortical regions, as well as between different cortical areas. Its role as a node of multiple brain pathways implicated in processing of sensorial inputs makes this brain region a critical hub for high-order cognition, as well as for emotion processing^35^. Our results show that the combination of caudate-thalamus-MI_TG regions are common between SZ and MDD, which associate with both PANSS negative dimensions related to depression (blunted affect, emotional withdrawal and stereotyped thinking) and poorer cognition (reasoning) in SZ. Evidence had previously suggested that there is an overlap between certain negative symptoms of schizophrenia and depressive symptoms^36^. Middle temporal cortex dysfunction was observed in clinical high-risk individuals in psychosis^37^. It appears that the temporal cortex structural and functional abnormality underly auditory hallucinations in schizophrenia^38^. However, our results indicate that MI_TG combined with caudate and thalamus together are associated with comorbidity between depression and SZ, which contribute to both PANSS negative symptoms and cognition in SZ. Patients with schizophrenia are at an increased risk for the development of depression^36^. Overlap in brain imaging abnormalities in caudate-thalamus-MI_TG between the two disorders suggests a common patho-physiological mechanism including both symptom and cognition may underlie the presentation of comorbid depression in schizophrenia. Hence, we hypothesize that depression may contribute to some of the negative symptoms and cognitive deficits seen in SZ through a network involving the caudate-thalamus-MI_TG. However, this hypothesis would better be examined in a longitudinal MRI/rest fMRI study in SZ patients with persistent negative symptoms treated with antidepressant medication. We would expect that those depressed SZ who have a normalization in the caudate-thalamus-MI_TG network would be most likely to benefit from the treatment.

ITG in GMV represent the common brain network among SZ, ADHD, and ASD. ITG is the most consistent findings related to many of the behavioral deficits observed in individuals with ASD, such as receptive language and social cognition^39,40^. Increased GMV in ITG was observed in both high functioning and low functioning autism^41^. Parietal–temporal involvement is apparent during executive function tasks that draw upon visual–spatial processes in working memory and inhibitory control in ADHD^42^. While in SZ, decreased ITG connectivity in the language network in schizophrenia patients is associated with auditory verbal hallucinations^43,44^. In this study, we discovered that ITG in gray matter, a specific pattern shared between developmental disorders (ASD and ADHD) and SZ, may be involved in the production of certain symptoms such as poor attention, as well as cognitive deficits such as speed of processing and reasoning in SZ. Hence, we would hypothesize that developmental disorders may contribute to the PANSS general symptoms and cognitive deficits seen in SZ through a network involving ITG in GMV. This hypothesis can be tested in a longitudinal MRI study of childhood-onset schizophrenia (a subgroup with presumably more severe neurodevelopmental pathophysiology) treated with social skills training for persistent general symptoms and cognitive deficits. Individuals with greater improvement in general symptoms would be expected to have a more robust normalization on GMV in ITG. Hence, the brain networks derived from our current approach, may be developed as targets to test engagement of specific interventions in controlled trials.

A potential limitation is that the multimodal brain imaging data were collected from multiple sites. The lack of standardization across MRI acquisitions, inclusion criteria, and clinical assessments should be considered. Previous studies suggest that there are co-morbidities between substance abuse, MDD and ADHD^45,46^. Therefore, the identified brain abnormality patterns in schizophrenia and other disorders may not be entirely independent. However, these substance use subjects, MDD, ASD, and ADHD had no current or past history of SZ. And the SZ subjects had no current or past history of other psychiatric or neurological illness. Although this study used static brain function (fALFF) approaches, dynamic functional network connectivity matrices^28,47^ can also be used to capture both temporal and spatial co-alterations in a future study, which could provide an even richer understanding schizophrenia-specific effects among different mental disorders from the temporal perspective. Such an analysis pipeline could also be directly applied to study other mental disorder specific effects.

To the best of our knowledge, this is the first attempt to evaluate the relationship between SZ and other mental disorders by combing multimodal brain imaging data from SZ, drinking, smoking, MDD, ADHD and ASD in one study. According to our current results, we highlight that (1): substance use may relate with cognitive deficits (speed of processing and working memory) in schizophrenia through GMV in ACC-thalamus reward circuit; (2) depression was linked to PANSS negative dimensions (blunted affect, emotional withdrawal and stereotyped thinking) and cognition (reasoning) in SZ through a network involving caudate-thalamus-middle/inferior temporal gyrus in GMV; (3) developmental disorders may influence PANSS general (poor attention) and cognitive (speed of processing and reasoning) dimensions in SZ through GMV in ITG. In summary, we find there is imaging evidence for the common underlying neural mechanisms between SZ and other mental disorders, and different disorders have potential different effects in SZ with respect to symptoms and cognition. Finally, these results may have heuristic value and suggest specific approaches to refine available treatment strategies for SZ concurrent with other disorders.

## Supplementary information

supplementary file

## Data Availability

The supervised fusion code has been released and integrated in the Fusion ICA Toolbox (FIT, https://trendscenter.org/software/fit), which can be downloaded and used directly by users worldwide. The SZ, MDD, drinking and smoking data can be accessed upon request to the corresponding authors. The ASD and ADHD data can be accessed upon application from ABIDE consortium and ADHD-200.
