# Supplementary material for: The relevance of transdiagnostic shared networks to the severity of symptoms and cognitive deficits in schizophrenia: a multimodal brain imaging fusion study
Source: Transl Psychiatry. 2020 May 18;10:149. doi: 10.1038/s41398-020-0834-6 (PMC7235018; doi:10.1038/s41398-020-0834-6)
Supplement: Supplementary file 1 — supplementary file [file 41398_2020_834_MOESM1_ESM.docx]

**Supplementary information**


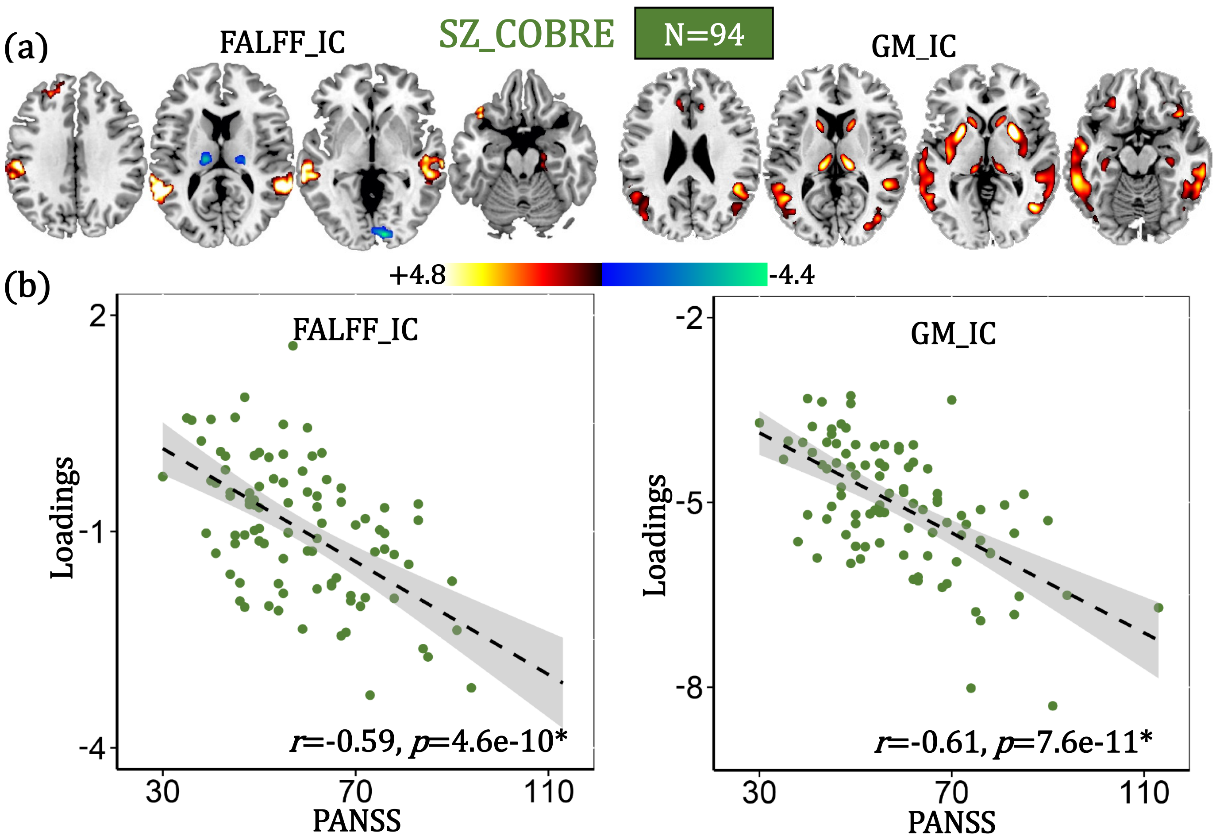


**Supplementary Figure 1**. The identified PANSS associated multimodal joint components in COBRE SZ group. (a) Spatial brain maps visualized at |Z| > 2. (b) Correlation scatter plot between PANSS scores and loadings of component for each modality. The gray areas in (b) indicate a 95% confidence interval.


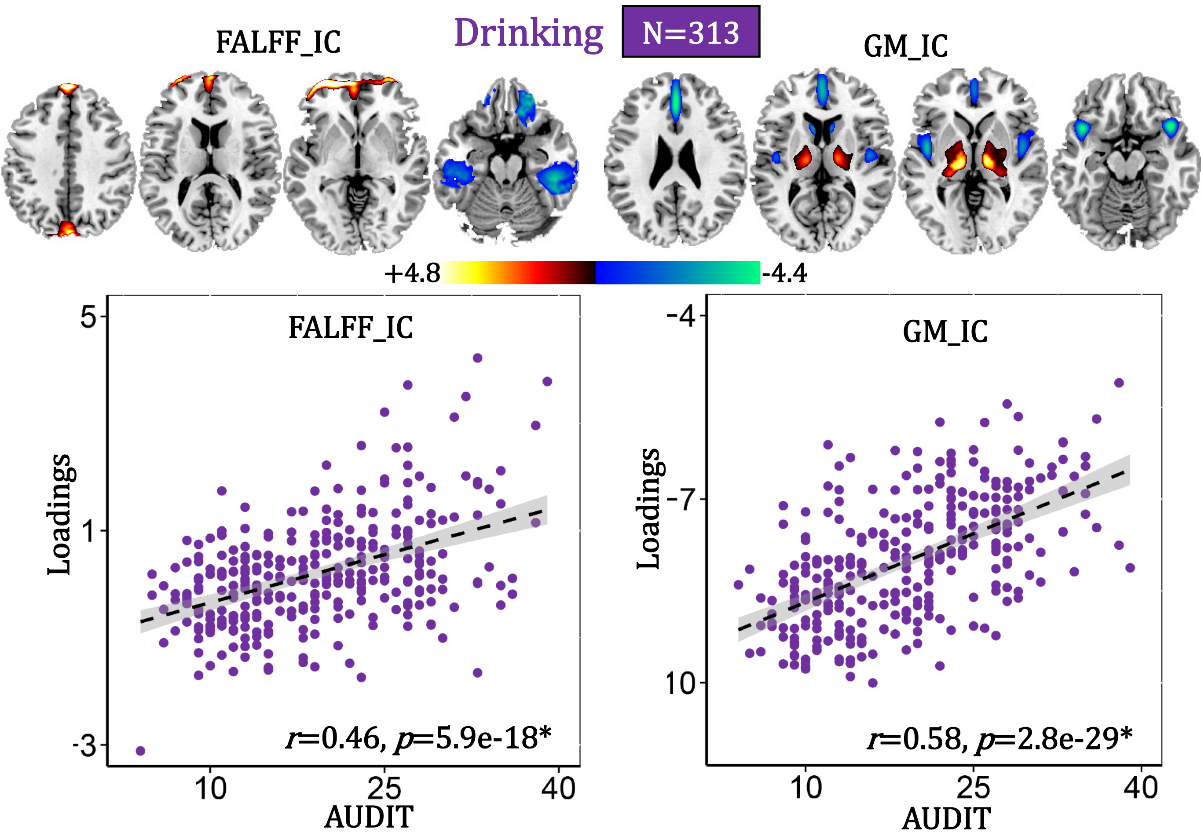


**Supplementary Figure 2**. The identified AUDIT associated multimodal joint components in drinking group.


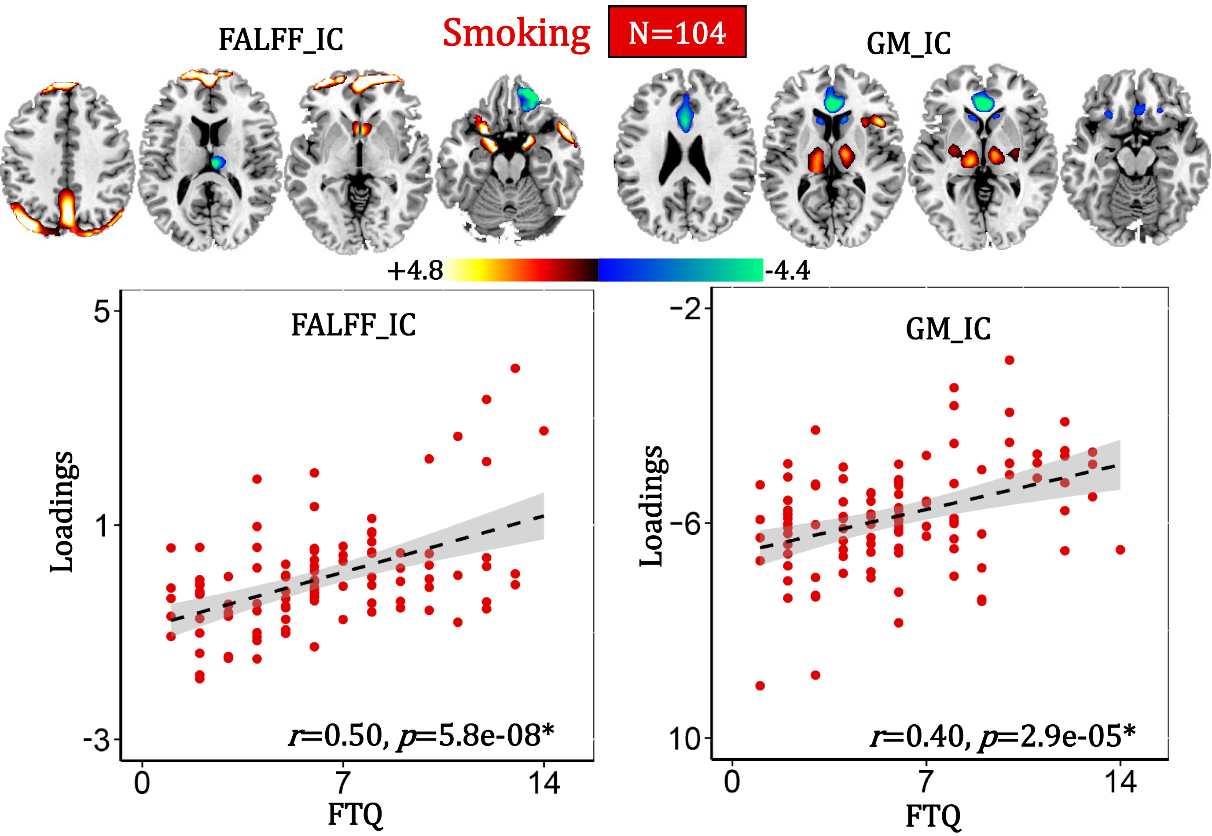


**Supplementary Figure 3**. The identified FTQ associated multimodal joint components in smoking group.


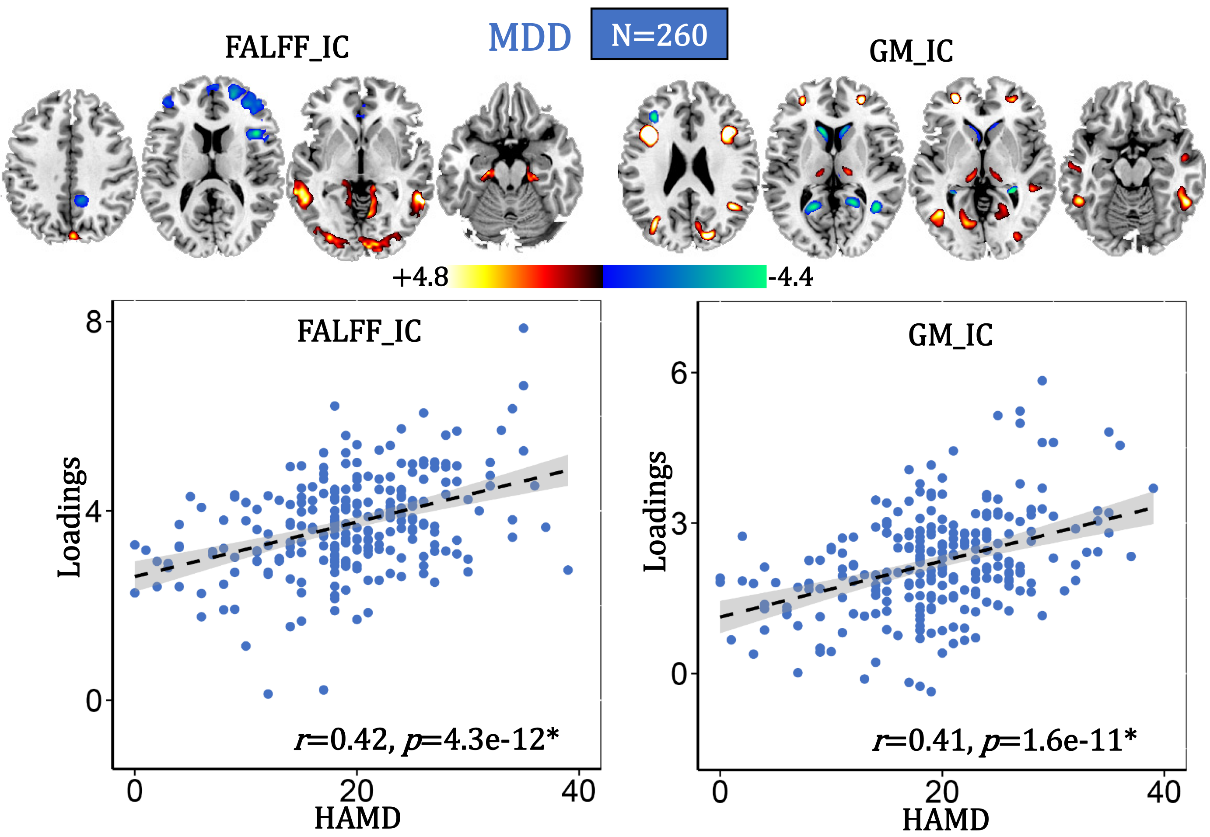


**Supplementary Figure 4**. The identified HAMD associated multimodal joint components in MDD group.


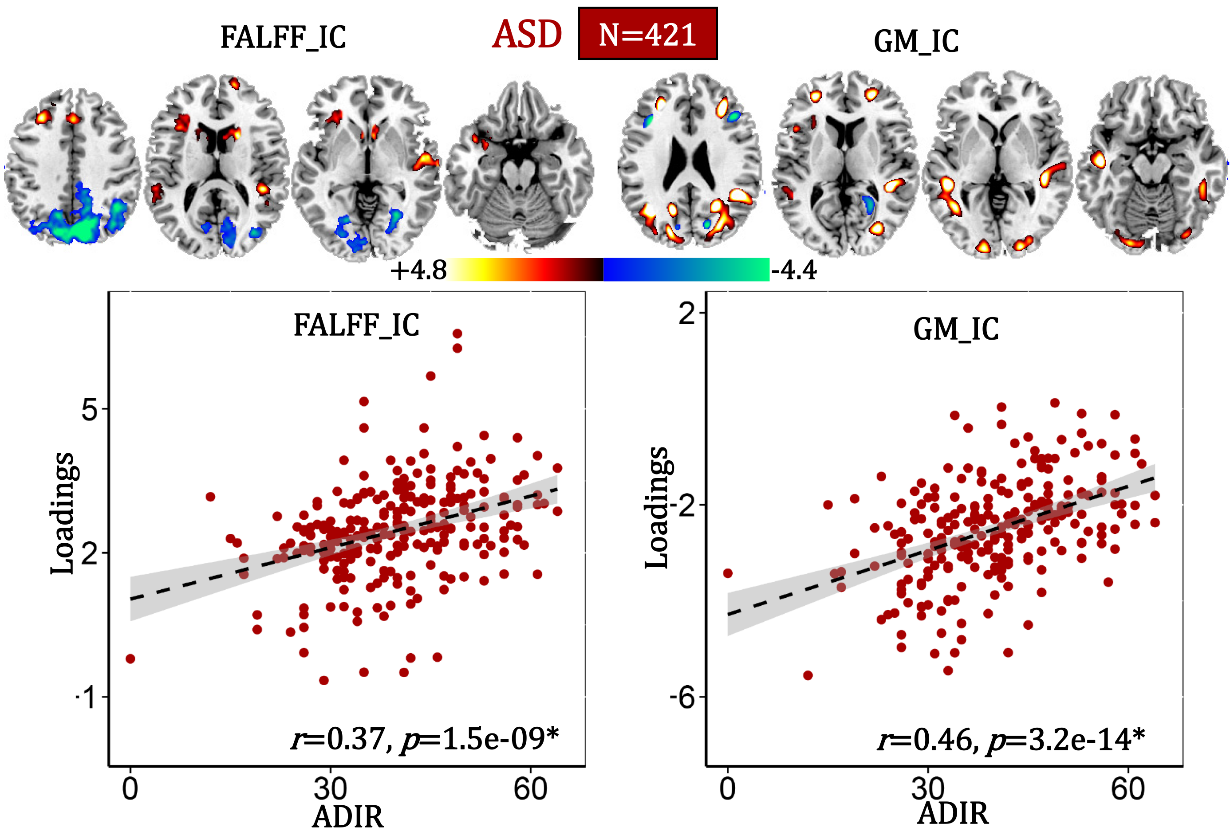


**Supplementary Figure 5**. The identified ADIR associated multimodal joint components in ASD group.


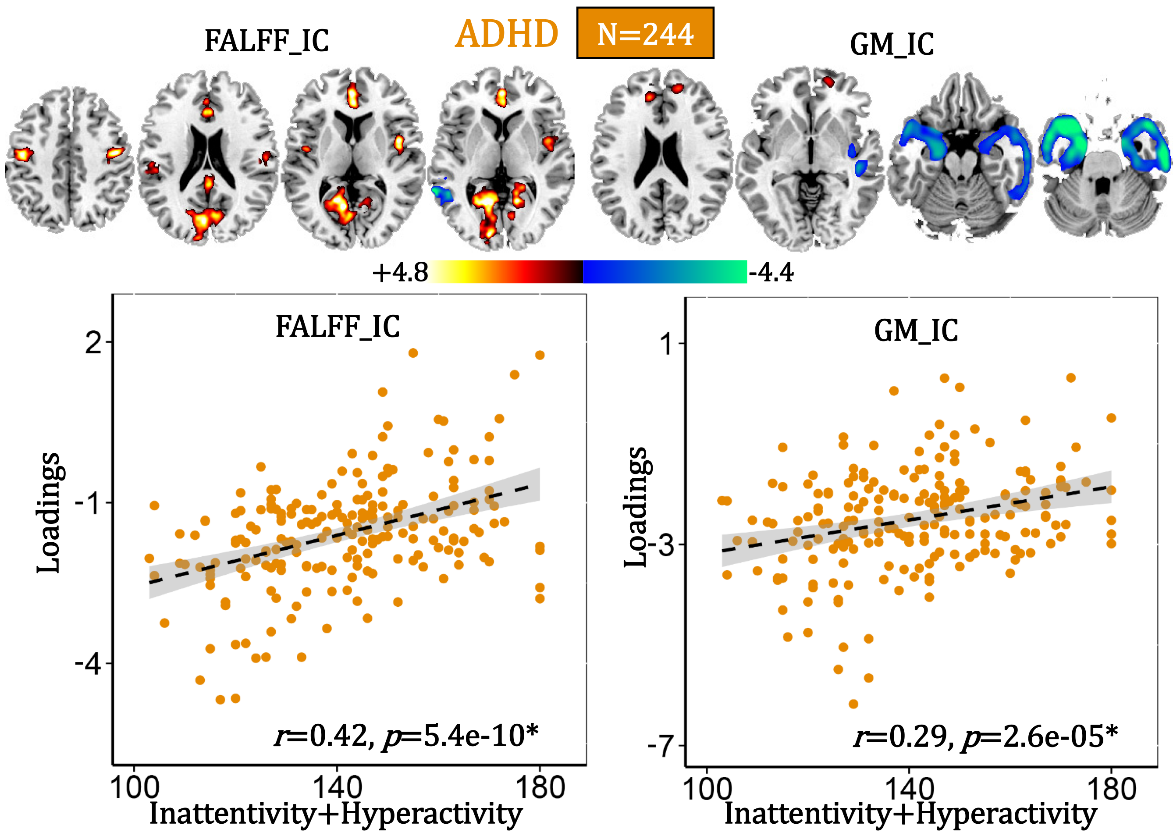


**Supplementary Figure 6**. The identified ADHD symptom total score associated multimodal joint components in ADHD group.
